# Diurnal and day-to-day movement patterns of finishing pigs on deep straw bedding during the last 20 d before slaughter

**DOI:** 10.1093/jas/skag122

**Published:** 2026-04-08

**Authors:** Marko Ocepek

**Affiliations:** Faculty of Biosciences, Department of Animal and Aquacultural Sciences, Norwegian University of Life Sciences, Ås, 1432 Ås, Norway

**Keywords:** deep straw, diurnal activity, environmental factors, finishing pigs, individual variation, precision livestock farming

## Abstract

Monitoring movement patterns of pigs provides valuable insight into welfare, health, and adaptation to housing environments, yet individual- and time-resolved evidence from deep-straw systems is limited. We monitored 16 finishing pigs housed on deep straw bedding during the last 20 d before slaughter using an RFID (radio-frequency identification)–LoRaWAN (Long Range Wide Area Network) system. One-Hz detections were converted to estimated distance from the receiver and aggregated to hourly movement (cm·h^−1^). In total, 7,680 pig×day×hour records were possible (16 pigs × 20 d × 24 h); hours without valid positioning data were excluded, resulting in 6,026 records for inferential analyses. Hourly movement was analyzed using a linear mixed model with fixed effects of hour, day, and their interaction, a random intercept for pig, and an AR(1) residual structure. Pigs exhibited a clear diurnal rhythm with movement lowest during the night and peaking between midday and afternoon. Movement differed among days, and the day × hour interaction showed day-specific modulation of the diurnal profile. Between-pig differences were substantial (ICC ≈ 0.29), supporting the use of individual baselines for precision monitoring. Environmental covariates (temperature, relative humidity, PM2.5) were not associated with movement in the day-by-timeline model. These results highlight the value of hour-aware and individualized approaches when using movement data for precision livestock farming applications in enriched housing.

## Introduction

Monitoring activity and movement in finishing pigs can provide practical insight into welfare, health, and how animals adapt to their housing environment. Movement is a coarse but continuous indicator of activity and space use; sustained deviations from an individual’s typical pattern may signal discomfort, illness, or management-related disturbances before clear clinical signs are observed. Recent progress in precision livestock farming (PLF) enables continuous monitoring under commercial conditions using cameras and computer vision (Ocepek et al 2021) as well as sensor-based systems, supporting early warning and decision support in pig production (Kashiha et al 2013; Nasirahmadi et al 2016; Guo et al 2023; Yang et al 2024; Li et al 2024; Jiang et al 2025; Decarie et al 2025).

Daily (diurnal) rhythms in pig activity have been described in a range of production systems, but much of the available evidence relies on pen- or group-level summaries rather than continuous, individual time series (Simonsen 1990). In commercial pens, pigs cannot always access key resources simultaneously, and competition at feeders can alter within-day patterns and timing of visits, effectively spreading activity across the day (Hyun and Ellis 2002; Durand et al 2023). Greater space allowance and enriched floor substrates such as straw bedding may further increase locomotion and exploration beyond resource-directed movement, which could dilute strict synchrony in group averages and accentuate individual differences. Recent work using automated systems also shows consistent between-pig differences in diurnal and circadian patterns (including night feeding), suggesting that group averages may mask meaningful individual strategies that may relate to social status (Bus et al 2023; Bus et al 2024; Ochoteco-Asensio et al 2024). Consequently, individual- and hour-resolved evidence from deep-straw finishing systems during the final weeks before slaughter remains limited.

Deep-straw finishing systems are relevant to welfare because they provide substrate for exploration and manipulation and are aligned with requirements and recommendations for enrichment and bedding in several regulatory and welfare frameworks (Lovdata 2003). Enrichment and access to rooting materials can influence behavioral expression and welfare outcomes and can change how pigs allocate activity across time and space (Ocepek et al 2020; Andersen et al 2023; Ocepek et al 2025). Despite frequent on-farm impressions that pigs may appear less active late in the finishing period, systematic quantification of day-to-day and hour-to-hour movement dynamics during the last weeks before slaughter remains limited.

Indoor environmental conditions, particularly temperature and relative humidity, are expected to influence activity and welfare. As temperature increases, pigs typically reduce locomotion and shift time budgets toward resting and heat-dissipating behaviors; higher humidity can exacerbate heat load by impairing heat loss and may lower the temperature threshold at which behavioral changes occur (Huynh et al 2005). Air quality indicators such as particulate matter (PM; PM1, PM2.5, and PM10, representing particles with aerodynamic diameter ≤1, 2.5, and 10 µm) are also relevant because higher dust loads can irritate the respiratory tract and contribute to inflammation, potentially affecting comfort and longer-term activity (Ocepek and Škorjanc 2016; Ocepek and Andersen 2022). Under commercial conditions, however, environmental variation may be modest relative to strong temporal rhythms, and environmental measurements are often available at coarser temporal resolution than behavioral data, complicating inference about environmental contributions.

The objectives of this study were to use a radio-frequency identification (RFID) positioning system with Long Range Wide Area Network (LoRaWAN) communication (RFID–LoRaWAN) to quantify movement patterns of finishing pigs housed on deep straw bedding during the last 20 d before slaughter, focusing on 1) group-level diurnal variation across the 24-h cycle, 2) systematic day-to-day variation across the finishing period, including day-by-hour modulation of the diurnal profile, 3) persistent between-pig differences in movement levels, and 4) associations between movement and indoor environmental conditions (temperature, relative humidity, and PM).

Beyond group-level rhythms, individual pigs may differ in both baseline movement and in the timing of their activity. Such differences can arise from stable behavioral tendencies, but also from social dynamics: in group-housed pigs, access to preferred resources can be socially mediated, and subordinate individuals may shift activity to less competitive periods, resulting in weaker synchrony and individual-specific daily profiles. In addition, illness or subclinical discomfort is expected to reduce movement and disrupt an individual’s typical pattern, implying that longitudinal monitoring could support earlier detection in systems with larger groups where individual visual inspection is more challenging.

We predicted that pigs would show a clear diurnal rhythm with a midday to afternoon peak; that movement would vary among days and among individuals; and that the hourly profile would differ among days (day × hour interaction), reflecting day-specific modulation of the diurnal profile. We further expected persistent between-pig differences consistent with individual baselines, potentially including shifts in activity timing consistent with social resource competition (e.g., subordinate pigs being relatively more active outside peak periods). Finally, we predicted that environmental variables would explain only a small proportion of variation under the relatively moderate indoor conditions of the study, with stronger temperature-related effects expected under higher heat load. We also expected that a sick individual would show consistently reduced movement relative to its typical pattern, illustrating the potential of continuous monitoring for earlier detection in commercial settings.

## Material and methods

### Ethical considerations

The study complied with Norwegian animal welfare legislation (Lovdata 2003; Lovdata 2013). All observations were conducted on-farm under standard commercial management routines and involved no experimental treatments or manipulations.

### Animals and housing

**Figure 1:**
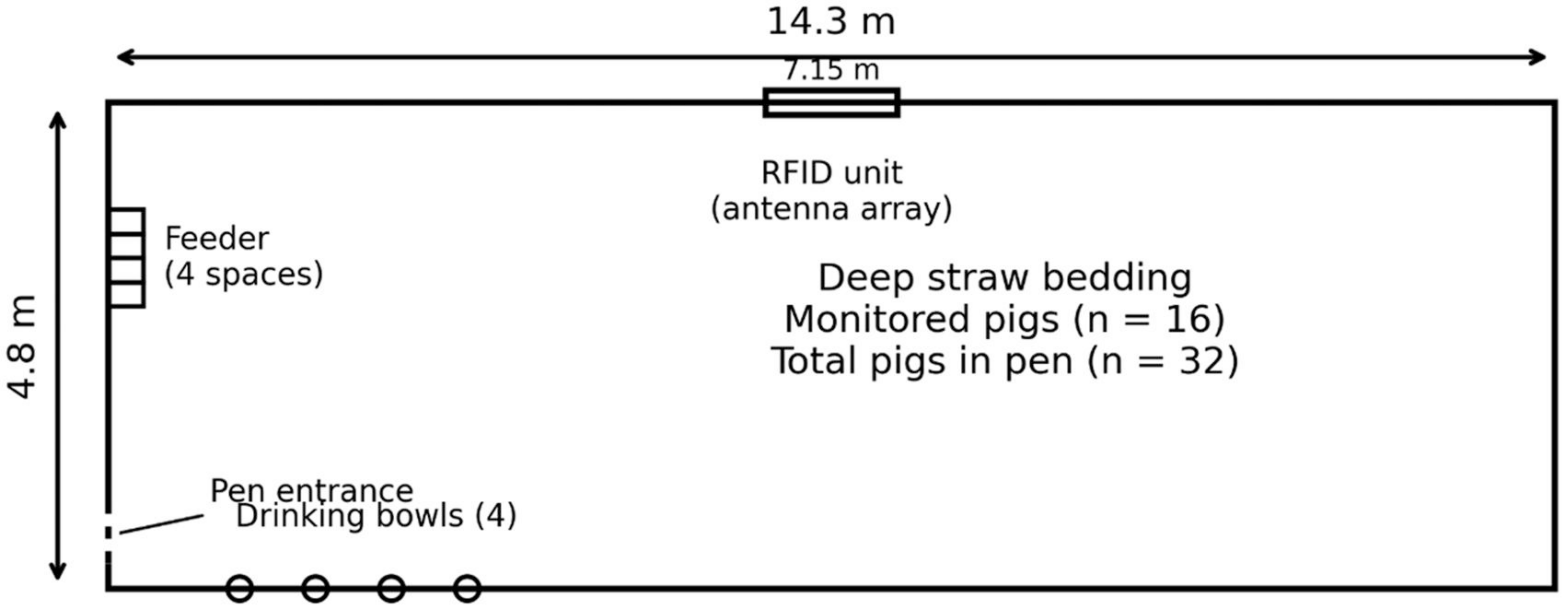
Schematic layout of the deep-straw finishing pig pen (14.3 × 4.8 m) used in the study. The pen housed 32 pigs, of which 16 pigs were fitted with passive RFID ear tags and individually monitored. The pen entrance and a four-space dry feeder were located on the short wall. Four drinking bowls (ad libitum water) were located along the adjacent long wall. The RFID unit (antenna array) was mounted midway along the opposite long wall (7.15 m to each corner), providing approximately equal coverage (∼7 m) toward both ends of the pen. Pigs were kept on deep straw bedding (straw replenished weekly, one bale) under standard farm routines with continuous access to manipulable material. The barn contained three identical pens.

The study was carried out in a Norwegian commercial deep-straw finishing pig unit during the final 20 d before slaughter. Data were collected from February 2 to February 21, 2023 (Day 1–20). The pigs represented a Norwegian commercial fattening pig genotype, consisting of TN70 crossbred sows (Norsvin Landrace × Topigs Norsvin Z-line) inseminated with Norsvin Duroc boar semen. The room comprised three identical deep-straw pens, each housing 32 finishing pigs. Data collection was conducted in one pen (14.3 m × 4.8 m; Figure 1); within this pen, 16 of the 32 pigs were equipped with passive RFID ear tags and individually monitored throughout the study period. The monitored animals were of mixed sex (eight barrows and eight gilts). The pen had a solid concrete floor covered with deep straw bedding, which was replenished weekly (one straw bale). The pen contained a four-space dry feeder, and four drinking bowls were located on the left wall adjacent to the feeding area (as viewed from the corridor). The RFID receiver cabinet/antennas were mounted at the midpoint of the right-hand wall of the monitored pen (Figure 1), providing an effective detection coverage of approximately 7.15 m in each direction along the pen’s long axis (i.e., full coverage of the 14.3 m pen length). Animals were managed according to the farm’s standard routines and were not subjected to experimental treatments.

### Sensor system, calibration, and movement data processing

Pig movement was monitored using a custom RFID-based sensor network installed in the barn (Figure 1). Fixed receivers and external antennae were positioned around the monitored (wall-side) pen area and connected to a central cabinet containing the power supply, receiver units, and networking components. Receivers communicated via Wi-Fi and LoRaWAN (via a MikroTik gateway) and logged time-stamped detections with receiver identity and received signal strength indicator (RSSI). Data were transferred automatically to a local server and stored as event-based records with time stamps (typical inter-detection interval ∼3–5 s, depending on tag orientation and proximity to antennae).

Calibration and validation were performed before data collection by moving tagged transponders along known distances and locations to verify detection consistency across receivers. A pilot calibration dataset was used to convert RSSI to distance (m) using an empirical log-distance model. Specifically, distance (m) was estimated as:


distance=10 ((T×Power-RSSI)/(10×n)),

where *T*×Power is the assumed received signal strength (dBm) at 1 m and n is the path-loss exponent. *T*×Power and *n* were determined from the calibration measurements and then applied to the main dataset to obtain distance-to-antenna estimates. Because RSSI-based distances are sensitive to orientation and multipath effects, particularly at longer ranges, calibration and system testing were used to confirm that distance changes corresponded to known movements under barn conditions.

Movement was quantified from consecutive distance estimates. For each pig, the incremental distance between two consecutive detections was computed as the absolute change in estimated distance (e.g., 4 m → 2 m → 5 m corresponds to 5 m moved over that sequence). Incremental distances were summed within each hour to obtain hourly distance moved (m·h^−1^); values are reported in cm·h^−1^ in Results and Figures. Hours without valid positioning data (i.e., no valid detections to estimate distance) were excluded from inferential analyses; consequently, pigs contributed different numbers of valid hourly observations (Table S1). Importantly, hours without valid positioning data reflect lack of positioning information rather than confirmed inactivity, whereas near-zero movement values indicate low estimated locomotion within the monitored coverage zone during hours with valid detections.

All movement (and environmental) data used in this study were collected from the monitored pen only. Although the antenna range covered the full pen length, detections were most reliable in the wall-side zone closest to the antenna; therefore, the movement metric primarily reflects locomotion within the monitored coverage zone and may underrepresent activity occurring predominantly on the opposite side of the pen (e.g., near drinkers). This spatial limitation should be considered when interpreting absolute movement values (Figure 1).

### Environmental measurements

Environmental conditions were recorded in the same barn using stationary sensors placed at animal level within the monitored pen. Measurements included air temperature (°C), relative humidity (%), and particulate matter concentrations (PM1, PM2.5, PM10; µg·m^−^³), defined as mass concentrations of particles with aerodynamic diameters ≤1, 2.5, and 10 µm, respectively. Environmental data were recorded repeatedly within day (approximately 80–97 observations per day) and summarized to a day × Timeline level by calculating mean values per day and time block. Timeline blocks were defined as: Timeline 1 (00:00–07:00), Timeline 2 (08:00–15:00), and Timeline 3 (16:00–23:00).

### Statistical analysis

Hourly distance moved was analyzed using a linear mixed-effects model (PROC MIXED in SAS 9.4; SAS Institute Inc., Cary, NC, USA) with restricted maximum likelihood (REML) estimation. Fixed effects included the day of the finishing period (Day_s; days 1–20), the hour of day (0–23 h), and their interaction (Day_s × hour). Pig identity (pig_id) was included as a random intercept to account for repeated measures on the same animal. To model residual autocorrelation among consecutive hourly observations within each pig on the same day, an autoregressive covariance structure of order 1 [AR(1)] was applied (i.e., assuming measurements 1 h apart within a pig-day are correlated). Denominator degrees of freedom were determined using the containment method. Statistical significance was declared at *P* < 0.05.

The linear mixed-effects model can be expressed as:


$$y_{\mathrm{idh}}=\mu +{\text{Day}}_{d}+{\text{Hour}}_{h}+(\text{Day}\times \text{Hour}{)}_{dh}+b_{i}+\varepsilon_{\mathrm{idh}},$$

where $y_{\mathrm{idh}}$denotes hourly distance moved (cm·h^−1^) for pig *i* on day *d* at hour *h*, μ is the overall intercept, Day and Hour are fixed effects of day (1–20) and hour of day (0–23), respectively, and Day × Hour represents their interaction. The term $b_{i}$is a random intercept for pig *i* assumed to follow a normal distribution with mean 0 and variance $\sigma_{\text{pig}}^{2}$. The residual term $\varepsilon_{\mathrm{idh}}$represents within-pig unexplained variation and was modeled using a first-order autoregressive [AR(1)] covariance structure to account for temporal autocorrelation among successive hourly observations within pig and day.

Environmental sensor data were recorded at high frequency throughout each day (approximately 80–97 measurements per day) and were aggregated into three-time blocks per day, defined as Timeline 1 (00:00–07:00), Timeline 2 (08:00–15:00), and Timeline 3 (16:00–23:00). For each day × Timeline combination, mean values of temperature (°C), relative humidity (%), and particulate matter concentrations (PM1, PM2.5, and PM10; µg·m^−^³) were calculated. Because PM1, PM2.5, and PM10 were highly correlated (Pearson *r* = 0.97–0.99), only PM2.5 was retained for subsequent modeling. Pig movement data were aggregated to the same temporal resolution by calculating the mean distance moved per hour for each pig within each day × Timeline (dist_mean, cm·h^−1^), and the number of hours contributing to each mean was recorded (n_hours). To evaluate associations between movement and environmental conditions, a second linear mixed model (PROC MIXED, SAS 9.4) was fitted with dist_mean as the dependent variable. Fixed effects included day (Day_s), Timeline, and the continuous environmental covariates (mean temperature, relative humidity, and PM2.5). Random intercepts were included for pig (pig_id) and day-by-Timeline (DayTL). Observations were weighted by n_hours. Denominator degrees of freedom were based on the containment method, and effects were considered statistically significant at *P* < 0.05.

## Results

### Descriptive statistics

Hourly movement data were collected from 16 finishing pigs over the last 20 d of the production period. The RFID–LoRaWAN positioning system generated more than one million raw antenna detection records, which were converted into an estimate of movement based on changes in antenna detection zones. For inferential analyses, raw detections were aggregated to an hourly movement measure (cm·h^−1^) for each pig. After exclusion of hours without valid positioning data, the final dataset comprised 6,026 pig × day × hour observations.

Across all pigs, days, and hours, mean (±SE) movement was 792.8 ± 3.2 cm·h^−1^ (7.93 m·h^−1^; SD = 249.9 cm·h^−1^), ranging from 0 to 1,439.9 cm·h^−1^ (Table 1). The interquartile range was 634.3–977.6 cm·h^−1^, and zero values occurred for all pigs, indicating hours with no detected movement within the monitored area.

**Table 1:** Descriptive statistics for hourly movement and environmental conditions during the observation period.

Variable	*n*	Mean	SE	Min	Max
**Hourly movement, cm·h^−1^**	6,026	792.8	3.2	0.0	1,439.9
**Interquartile range, cm·h^−1^**		634.3			977.5
**Temperature, °C**	1,507	16.05	0.04	8.35	19.48
**Relative humidity, %**	1,507	71.05	0.12	59.85	82.20
**PM1, µg·m^−3^**	1,507	6.37	0.15	0.00	22.00
**PM2.5, µg·m^−3^**	1,507	9.76	0.23	0.00	36.82
**PM10, µg·m^−3^**	1,507	14.54	0.29	1.32	50.46

Environmental summaries are weighted by the number of sensor records contributing to each day × timeline block (46 blocks; 1,507 total records). PM1, PM2.5, and PM10 denote particulate matter with aerodynamic diameter ≤1, ≤2.5, and ≤10 µm, respectively. Hourly movement was derived from changes in antenna detection zones; values of 0 indicate no detected movement within the monitored area during that hour.

Environmental conditions were summarized by day × Timeline blocks (46 blocks; 1,507 underlying sensor records) and showed relatively stable, moderate conditions during the period with available environmental data (Table 1). Weighted mean (±SE) temperature and relative humidity were 16.07 ± 0.04°C and 71.06 ± 0.12%, respectively. Mean (±SE) concentrations of airborne particulate matter were 6.37 ± 0.15 µg·m^−^³ (PM_1_), 9.76 ± 0.23 µg·m^−^³ (PM_2.5_), and 14.54 ± 0.29 µg·m^−^³ (PM_10_). The three PM fractions were highly correlated (Pearson *r* = 0.97–0.99); therefore, only PM_2.5_ was retained for subsequent modeling to avoid multicollinearity.

### Overall effects

Hourly movement (cm·h^−1^) was analyzed using PROC MIXED (REML estimation; degrees of freedom method: containment) with fixed effects of day (1–20), hour of day (0–23), and their interaction. Pig was included as a random intercept, and repeated hourly measurements were modeled using an AR(1) residual correlation structure within pig × day (hour repeated within pig × day). Overall effects are summarized in Table 2.

**Table 2:** Overall effects from the mixed model of hourly movement.

Effect	Num DF	Den DF	*P*-value
**Hour of day**	23	5560	<0.0001
**Day (d)**	19	5560	0.0013
**Day × hour**	437	5560	<0.0001

Type 3 tests of fixed effects from PROC MIXED (REML). Response variable: hourly movement (cm·h^−1^). Model note: pig was included as a random intercept; repeated measurements across hours were modeled using an AR(1) residual correlation structure within pig × day (hour repeated within pig × day). Estimated AR(1) parameter = 0.19.

**Figure 2:**
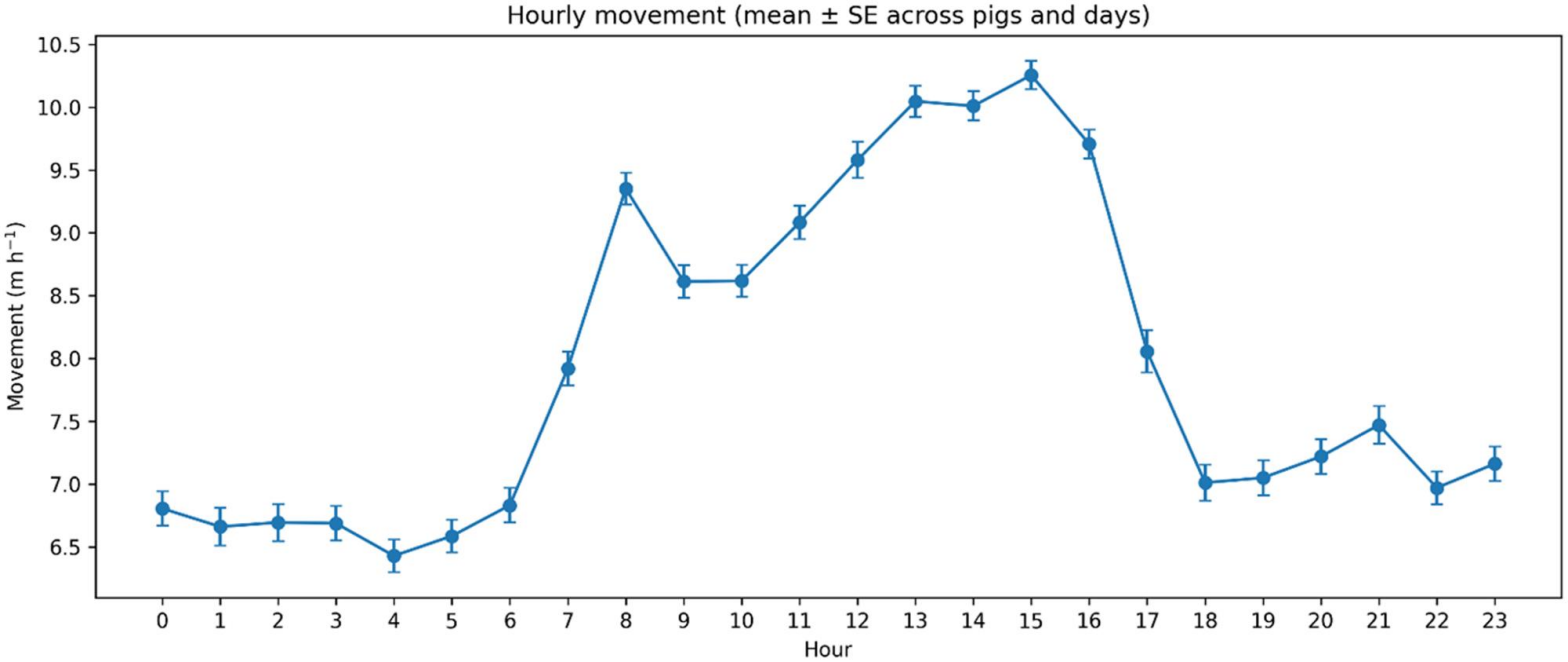
Least-squares means (± SE) of hourly movement (cm·h^−1^) across the 24 h cycle, averaged across pigs and days. Least-squares means were obtained from PROC MIXED (REML) with pig as a random intercept and an AR(1) residual correlation structure within pig × day (hour repeated within pig × day; estimated AR(1) = 0.19).

**Figure 3:**
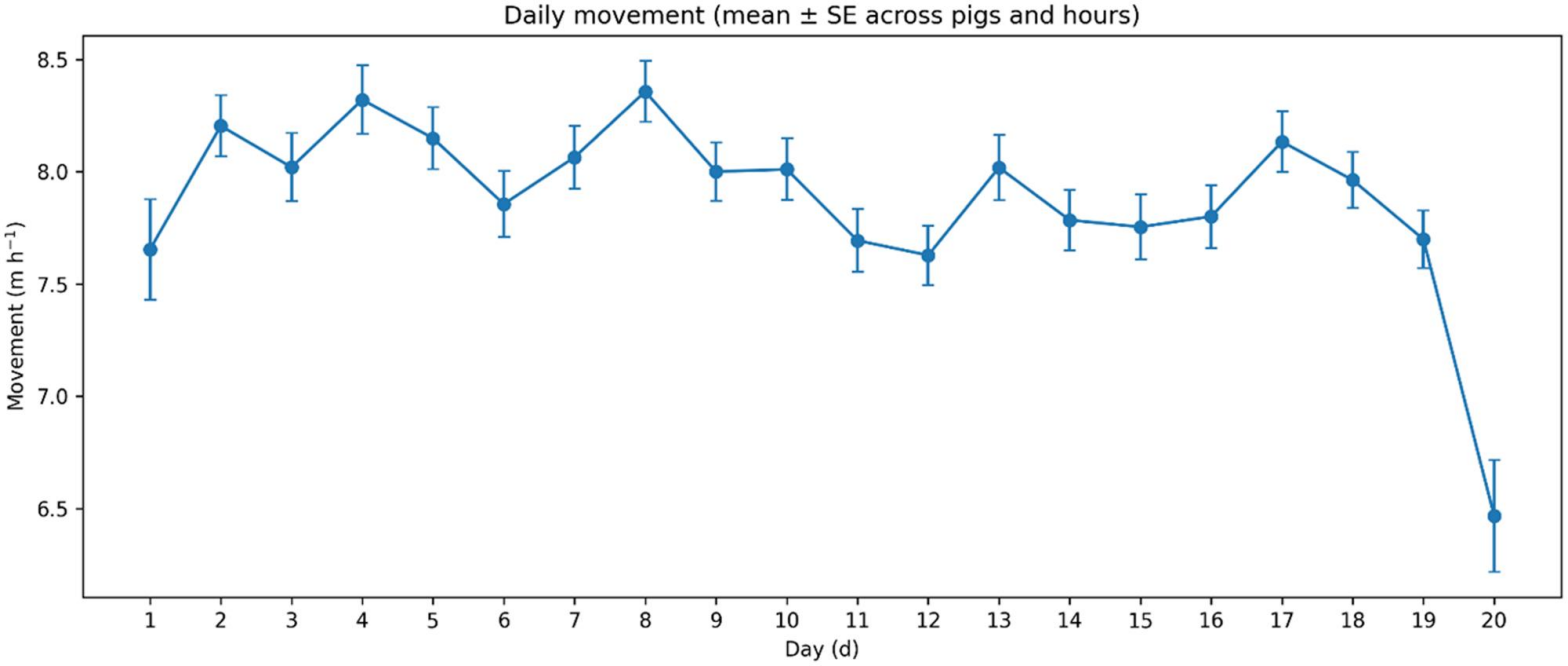
Least-squares means (± SE) of hourly movement (cm·h^−1^) by day (d), averaged across pigs and hours. Least-squares means were obtained from PROC MIXED (REML) with pig as a random intercept and an AR(1) residual correlation structure within pig × day (hour repeated within pig × day; estimated AR(1) = 0.19).

**Figure 4:**
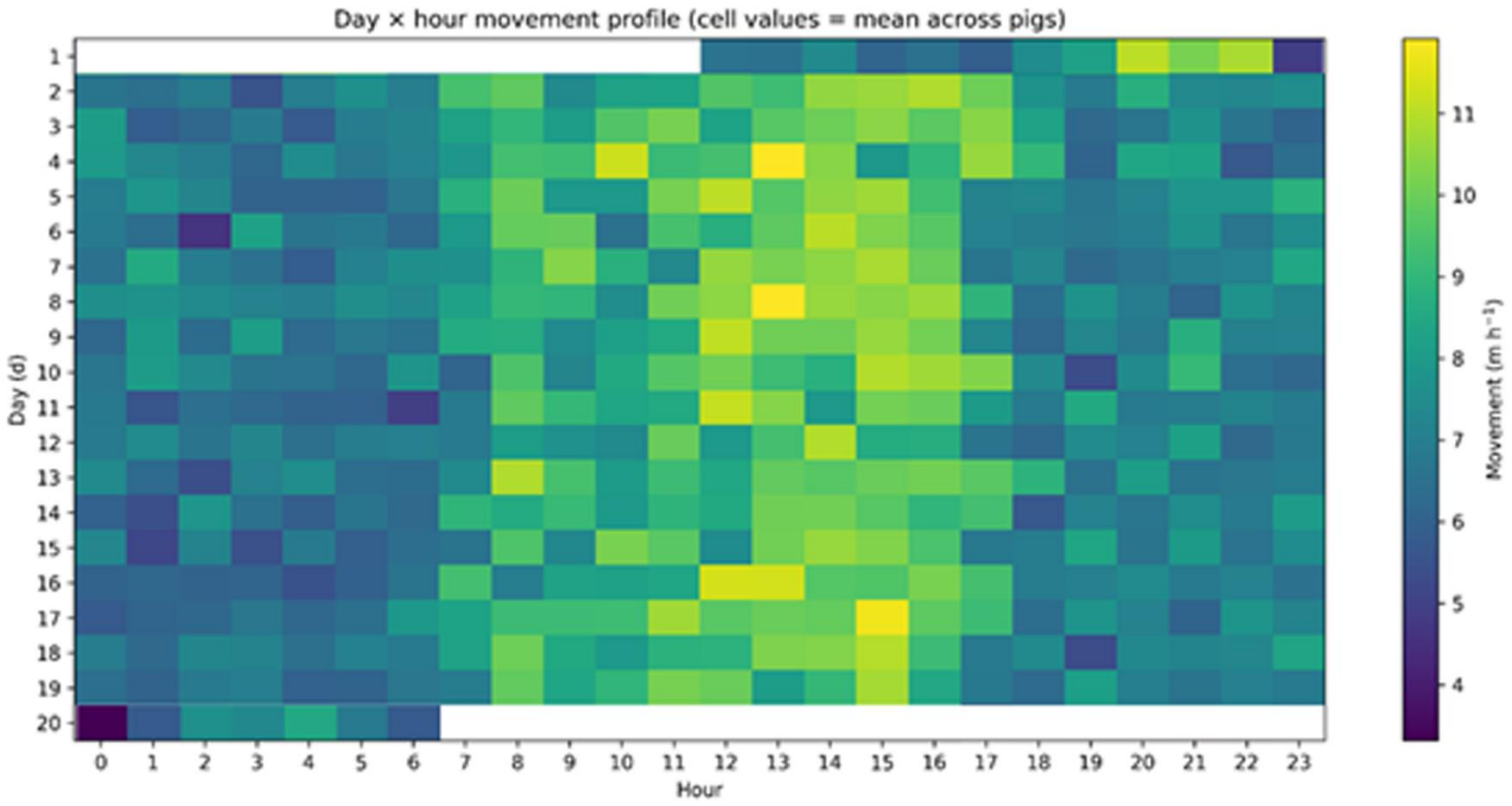
Least-squares means of hourly movement (cm·h^−1^) by day (d) and hour (day × hour interaction). Values are least-squares means from PROC MIXED (REML) with pig as a random intercept and an AR(1) residual correlation structure within pig × day (hour repeated within pig × day; estimated AR(1) = 0.19).

There were significant effects of hour (*P* < 0.0001) and day (*P* = 0.0013), and a significant day × hour interaction (*P* < 0.0001; Table 2). The estimated AR(1) parameter was 0.19, indicating weak positive autocorrelation among successive hourly observations within a pig-day. Across all pigs and days, least-squares (LS) mean movement varied by hour, being lowest at 01:00 (666.0 cm·h^−1^; 95% CI: 636.5–695.6) and highest at 15:00 (1,025.5 cm·h^−1^; 95% CI: 1,003.7–1,047.3) (Figure 2). Daily LSMeans (averaged across pigs and hours) differed across the observation period (Figure 3). The significant day × hour interaction reflected that the shape and/or magnitude of the hourly movement profile differed among days (Figure 4). Slice tests indicated significant differences among hours within each day (*P* < 0.0001, Table 2).

### Individual differences

**Figure 5:**
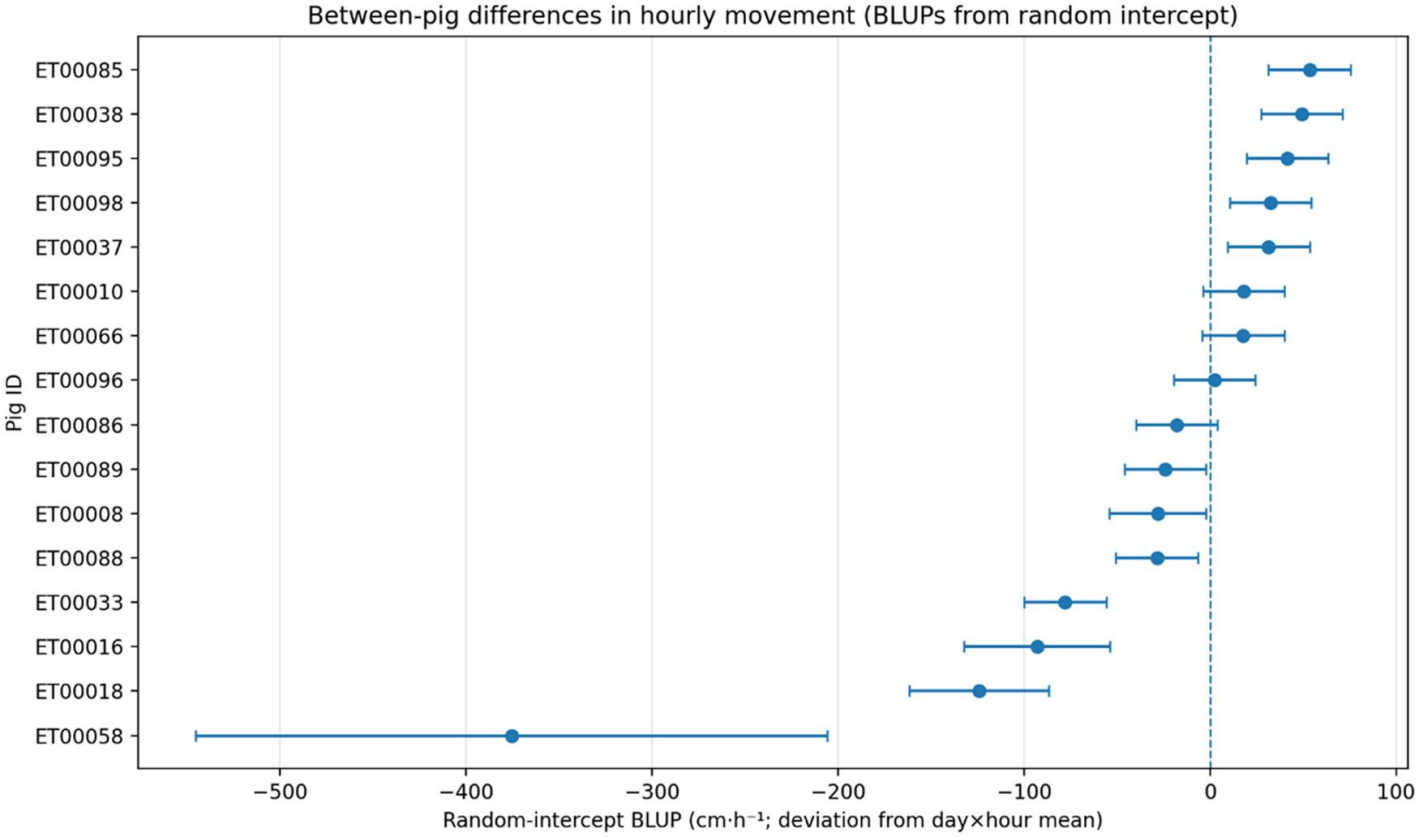
Pig-specific random-intercept BLUPs (±95% CI) for hourly movement from the mixed model (PROC MIXED; REML). Values represent each pig’s deviation (cm·h^−1^) from the fitted day × hour means. Pig was included as a random intercept; repeated measurements across hours were modeled using an AR(1) residual correlation structure within pig × day (hour repeated within pig × day; estimated AR(1) parameter = 0.19). The dashed line indicates zero deviation (overall mean pattern).

Between-pig variability was modeled using a random intercept for pig. The estimated variance of the pig random intercept was 15,773 (SD = 126 cm·h^−1^) and the residual variance was 38,884 (SD = 197 cm·h^−1^), corresponding to an intraclass correlation coefficient (ICC) of 0.29. Thus, after accounting for day, hour of day, and their interaction, approximately 29% of the remaining variation in hourly movement reflects persistent between-pig differences in baseline movement. On the original scale, the pig-level SD (126 cm·h^−1^; ∼1.3 m·h^−1^) indicates meaningful and stable differences in baseline movement among pigs, supporting the use of individualized baselines rather than a single group threshold. Pig-specific deviations from the overall diurnal pattern are illustrated using BLUPs (Figure 5). Most pigs clustered close to zero deviation, whereas ET00058 showed a pronounced negative deviation with wide uncertainty due to the very small number of available hourly observations. Pig ET00058 contributed only 4 hourly observations, resulting in an imprecise estimate (wide CI) and a large negative BLUP. BLUPs are conditional modes of the pig random intercept from the mixed model (units: cm·h^−1^); negative values indicate lower-than-average movement after accounting for day, hour, and day × hour. Figure 5 illustrates that, despite a shared overall diurnal pattern, pigs differed consistently in their baseline movement level, supporting the presence of stable individual differences beyond short-term fluctuations.

### Environmental effects on movement

Environmental covariates were evaluated as additional predictors of movement after accounting for the temporal structure identified previously (Day and Timeline). In the mixed model including Day, Timeline, and mean environmental measures (temperature, relative humidity, and PM2.5), both Day (*F*_18,603_=1.71, *P* = 0.0339) and Timeline (*F*_2,603_=157.96, *P* < 0.0001) were significant (Table 3). In contrast, temperature (*F*_1,603_=0.37, *P* = 0.5438), relative humidity (*F*_1,603_=0.07, *P* = 0.7974), and PM_2.5_ (*F*_1,603_=0.00, *P* = 0.9784) were not associated with movement (Table 3).

**Table 3:** Type 3 tests of fixed effects.

Effect	Den DF	*F* value	*P*-value
**Day, n**	603	1.71	0.0339
**Timeline**	603	157.96	<0.0001
**Temperature, mean**	603	0.37	0.5438
**Relative humidity, %**	603	0.07	0.7974
**PM_2.5_, mean**	603	0.00	0.9784

Model note: Pig was included as a random intercept; repeated measures were modeled within pig × day × timeline as specified in the analysis dataset (*N* = 668).

## Discussion

This study used continuous, individual movement monitoring in finishing pigs housed on deep straw bedding to address four objectives: 1) describe the 24-h (diurnal) movement profile, 2) quantify day-to-day changes during the last 20 d before slaughter, including day-specific modulation of the diurnal pattern, 3) assess persistent between-pig differences that motivate individual baselines, and 4) evaluate whether indoor environmental conditions (temperature, relative humidity, particulate matter) contribute to movement variation. Overall, the findings supported our predictions of a clear diurnal rhythm, meaningful day-to-day variation including modulation of the hourly profile, and substantial individual differences, whereas environmental covariates explained little additional variation under the relatively moderate conditions observed.

### Group-level diurnal rhythm in movement

Across pigs and days, movement followed a pronounced diurnal rhythm with lowest activity during the night/early morning and a clear peak from midday to afternoon. On average, movement during the midday to afternoon window (12:00–16:00) was approximately 46% higher than during the night/early morning period (00:00–06:00), and the single highest hour was about 56% higher than the lowest hour. This pattern differs from the more classical bimodal activity profiles often reported in conventional fully slatted systems, where peaks can be linked to discrete feeding- or management-related events (Stolba and Wood-Gush 1989; Simonsen 1990; Day et al 1995; Temple et al 2011). In a deep-straw environment with continuous access to manipulable bedding and ad libitum feeding, exploration and locomotor activity may be distributed more broadly across the light period, reducing tight synchrony around single events and yielding a more consolidated afternoon peak.

Because pigs cannot always access key resources simultaneously in commercial pens, competition at a four-space feeder can shift the timing of visits and spread activity across the day, contributing to less synchronized group-level profiles (Hyun and Ellis 2002; Durand et al 2023). In the present pen, pigs had access to four feeder spaces and four drinkers (eight access points for 32 pigs), representing relatively high resource availability compared with common commercial configurations. Higher resource availability in enriched housing may reduce competition at key resources, potentially allowing more synchronous group activity patterns and less temporal displacement of subordinate animals. This interpretation is consistent with field observations from enriched finishing systems reporting low levels of aggression around resources under higher resource availability (Ocepek et al 2025), although aggression and dominance were not quantified in the present dataset. Importantly, our metric quantifies locomotion within the monitored zone and does not identify specific behaviors; interpretations should therefore be framed in terms of movement/activity rather than behavioral time budgets.

### Day-to-day variation and day-specific modulation of the diurnal profile

Movement was not constant across the last 20 d before slaughter, indicating that late-finishing activity dynamics include meaningful day-to-day modulation rather than a fixed “steady state.” The difference between the most active and least active day corresponded to roughly a 21% lower daily mean at the low end (and conversely about 27% higher at the high end). Rather than supporting a simple expectation of an abrupt drop in activity immediately before slaughter, the observed pattern is more consistent with gradual and fluctuating changes across days, potentially reflecting increasing body mass, shifting motivation for exploration, and evolving group dynamics as pigs approach slaughter weight.

Crucially, day-to-day differences were not merely shifts in overall daily movement level: the shape and/or timing of the hourly profile also varied among days. In practical terms, this means that “normal” movement on a given day is best described not as a single daily mean but as a day-specific diurnal profile, where the amplitude of the midday peak and the degree of evening decline can differ from day to day. Future work could test for change points in daily baselines and evaluate whether departures from expected day-specific profiles have predictive value for welfare or health outcomes in commercial monitoring.

### Persistent individual differences and individual baselines

After accounting for time structure (hour, day, and their interplay), a substantial share of remaining variation reflected persistent between-pig differences (ICC ≈ 0.29). Expressed on the original scale, this corresponds to a standard deviation of approximately 126 cm·h^−1^ for the pig-level random effect, indicating substantial and persistent differences in baseline movement among individuals. Among pigs with robust data coverage, mean hourly movement ranged from approximately 641 to 848 cm·h^−1^, meaning the most active individuals moved about 32% more than the least active individuals. This supports the central premise of individualized precision monitoring: because absolute movement levels differ among pigs, deviations from an individual’s expected range are likely to be more informative than deviations from a single group average.

The dataset also illustrated how individual monitoring can flag biologically meaningful outliers. One pig with very limited hours with valid positioning data showed markedly lower movement (≈ 226 cm·h^−1^, around 71% below the overall mean), consistent with a severe short-term deviation that could plausibly arise from illness or compromised condition. However, hours without valid positioning data reflect lack of positioning information (e.g., tag performance or time spent outside the most reliable coverage zone) and should be interpreted separately from low movement estimates derived from valid detections; therefore, reduced movement cannot be attributed to a specific clinical cause in the present study. While we did not quantify dominance rank, individual differences in both baseline movement and timing could also be influenced by social dynamics, where subordinate pigs may shift activity to less competitive periods, potentially contributing to weaker within-group synchrony and individualized daily profiles (Bus et al 2023; Bus et al 2024; Ochoteco-Asensio et al 2024). Together, these results reinforce that hour-aware, pig-specific baselines are a logical foundation for anomaly detection in group housing.

### Environmental covariates and spatial interpretation of the movement metric

Temperature, relative humidity, and particulate matter contributed little to explaining movement variation beyond the strong temporal structure. A plausible reason is that indoor conditions during the available period were relatively moderate and stable (mean temperature ∼16°C; mean relative humidity ∼71%), and behavioral responses to the thermal environment may become clearly detectable primarily under wider gradients or heat-load episodes. This interpretation aligns with established evidence that increasing temperature reduces locomotion and shifts time budgets toward resting and heat dissipation, with humidity potentially exacerbating heat load by limiting effective heat loss (Huynh et al 2005). Under hotter conditions, particularly during summer or during cyclic heat stress, stronger behavioral shifts would be expected, including altered within-day activity distribution and reduced movement (de Oliveira et al 2023).

Particulate matter was included because higher dust loads can irritate the respiratory tract and contribute to inflammation, potentially affecting comfort and longer-term activity (Ocepek and Škorjanc 2016; Ocepek and Andersen 2022). However, if PM levels are low or show limited day-to-day variability, detecting movement associations is inherently challenging. A second consideration is temporal alignment: environmental data often represent averaged conditions over broader intervals than behavioral time series, which can dilute short-lived associations.

Interpretation of the movement metric also requires attention to data completeness and spatial coverage. Hours without valid positioning data should not be interpreted as inactivity; they indicate that movement could not be estimated for that hour, whereas very low movement values represent low estimated locomotion within the monitored zone. The receiver was mounted at the midpoint of the long wall, and calibration and on-site testing indicated a practical range of approximately 7.15 m in each direction along the pen’s long axis, corresponding to coverage of the full pen length. However, detections were most reliable in the wall-side zone closest to the receiver; therefore, activity concentrated on the opposite side of the pen may be underrepresented, and near-zero movement hours should be interpreted as no detected movement within the monitored zone rather than complete inactivity of the pig.

### Implications for precision monitoring and future work

Together, the strong diurnal rhythm, day-specific modulation of the hourly profile, and stable individual baselines indicate that monitoring systems should be both hour-aware and individualized. Practical alerting thresholds should incorporate expected diurnal variation and normal day-to-day modulation and should focus on sustained departures from an individual’s baseline rather than short-lived fluctuations. This approach is particularly relevant in commercial settings with larger groups where continuous individual observation is not feasible and early deviations may be missed without automated support. Future work should 1) further validate the movement metric against independent behavioral measures (e.g., video-based activity scoring or automated vision tracking), 2) collect environmental data at higher temporal resolution and/or under broader thermal and air-quality gradients to better match behavioral time series, and 3) evaluate whether deviations from expected individual profiles predict clinical health events or welfare outcomes under a wider range of commercial conditions.

## Conclusions

In conclusion, finishing pigs housed on deep straw bedding showed a pronounced diurnal movement rhythm with a mid-day to afternoon peak, meaningful day-to-day modulation, and substantial persistent between-pig differences supporting the use of individual baselines for precision monitoring. Day-specific modulation of the diurnal profile indicates that “normal” activity is best interpreted as an expected hourly pattern that can vary across days, rather than a single stable daily mean. Associations between movement and temperature, relative humidity, and particulate matter were not detected under the relatively moderate and stable indoor conditions of this study, but environmental effects may become more apparent under wider climatic gradients or heat-load episodes. Together, these findings emphasize that continuous monitoring should be both hour-aware and individualized to avoid over-interpreting short-term fluctuations and to improve decision support in enriched housing systems.

## Supplementary Material

skag122_Supplementary_Data

## Data Availability

The datasets generated and analyzed during the current study are not publicly available due to institutional restrictions, but are available from the corresponding author upon reasonable request.

## Abbreviations

AR(1): first-order autoregressive
BLUP: best linear unbiased prediction
ICC: intraclass correlation coefficient
LoRaWAN: Long Range Wide Area Network
PLF: precision livestock farming
PM_1_, PM_2_._5_, PM_10_: particulate matter
REML: restricted maximum likelihood
RFID: radio frequency identification
