# Supplementary material for: Diurnal and day-to-day movement patterns of finishing pigs on deep straw bedding during the last 20 d before slaughter
Source: J Anim Sci. 2026 Apr 8;104:skag122. doi: 10.1093/jas/skag122 (PMC13167798; doi:10.1093/jas/skag122)
Supplement: skag122_Supplementary_Data [file skag122_supplementary_data.docx]

**Supplementary Material**

Supplementary Table S1. Number of valid hourly movement observations per pig after excluding hours without valid positioning data (no valid detections).

| Pig ID | Max possible hours | Hours with valid positioning data (n) | Hours without valid positioning data (n) | Valid (%) |
| --- | --- | --- | --- | --- |
| ET00008 | 480 | 321 | 159 | 66.9 |
| ET00010 | 480 | 451 | 29 | 94.0 |
| ET00016 | 480 | 139 | 341 | 29.0 |
| ET00018 | 480 | 152 | 328 | 31.7 |
| ET00033 | 480 | 451 | 29 | 94.0 |
| ET00037 | 480 | 451 | 29 | 94.0 |
| ET00038 | 480 | 451 | 29 | 94.0 |
| ET00058 | 480 | 4 | 476 | 0.8 |
| ET00066 | 480 | 451 | 29 | 94.0 |
| ET00085 | 480 | 451 | 29 | 94.0 |
| ET00086 | 480 | 451 | 29 | 94.0 |
| ET00088 | 480 | 449 | 31 | 93.5 |
| ET00089 | 480 | 451 | 29 | 94.0 |
| ET00095 | 480 | 451 | 29 | 94.0 |
| ET00096 | 480 | 451 | 29 | 94.0 |
| ET00098 | 480 | 451 | 29 | 94.0 |

Note: Hours without valid positioning data indicate insufficient RFID detections to estimate movement and were excluded from analyses; they do not represent confirmed inactivity. Very low movement values refer to hours with valid detections and reflect low estimated locomotion within the monitored zone. Maximum possible observations per pig were 480 hours (20 d × 24 h).
